# Calmodulin Adopts an Extended Conformation when Interacting with L-Selectin in Membranes

**DOI:** 10.1371/journal.pone.0062861

**Published:** 2013-05-02

**Authors:** Wei Deng, John A. Putkey, Renhao Li

**Affiliations:** 1 Aflac Cancer and Blood Disorders Center, Department of Pediatrics, Emory University School of Medicine, Atlanta, Georgia, United States of America; 2 Department of Biochemistry and Molecular Biology, The University of Texas Health Science Center at Houston, Houston, Texas, United States of America; Institute of Molecular and Cell Biology, Singapore

## Abstract

Calmodulin, an intracellular calcium-binding protein, is thought to regulate ectodomain shedding of many membrane proteins, but the underlying molecular mechanism has remained unclear. Basing on a solution structure of calcium-loaded calmodulin in complex with a L-selectin fragment that contains a portion of its transmembrane domain, Gifford *et al.* (University of Calgary) recently suggested that calmodulin regulates L-selectin shedding by binding directly to a portion of the L-selectin transmembrane domain in a compact conformation. Using fluorescently labeled calmodulin, we show however that calmodulin adopts a distinctly different and much more extended conformation when it binds to the CLS peptide (*i.e.* the entire transmembrane and cytoplasmic domains of L-selectin) reconstituted in the phosphatidylcholine liposome with micromolar dissociation constant and in a calcium-independent manner. Calmodulin adopts a similarly extended conformation in a ternary complex with the N-terminal FERM domain of moesin and CLS reconstituted in the phospholipid liposome that mimics the native membrane environment. These results indicate that calmodulin does not bind directly to the transmembrane domain of L-selectin. Understanding the association of calmodulin with L-selectin helps to shed light on the mechanisms underlying regulation of ectodomain shedding.

## Introduction

Many membrane proteins undergo regulated proteolysis at a site close to the cell membrane [1]–[3]. This process, named ectodomain shedding, is employed by many types of cells to regulate the expression and function of their surface molecules and to modulate a diverse array of cellular and physiological activities [1], [4]. Alterations in ectodomain shedding often lead to diseases including cancer, neurodegenerative diseases, and various inflammatory disorders [5], [6]. Although the biochemical nature of ectodomain shedding has been largely elucidated, the underlying regulation mechanisms remain elusive.

L-selectin is one of the best characterized ectodomain shedding substrates. It is a type I transmembrane protein expressed mainly on the surface of leukocytes [7]. L-selectin mediates the initial tethering and subsequent rolling of circulating leukocytes on the surface of endothelial cells lining the blood vessel [8]–[12]. L-selectin is cleaved physiologically by ADAM17 at a peptide bond between Lys^283^ and Ser^284^ [13], [14]. Several studies have indicated that the cytoplasmic domain of L-selectin plays a critical role in shedding regulation, as mutations in this domain could modulate shedding of L-selectin [15]–[19]. Since the cytoplasmic domain of L-selectin contains only 17 residues, the effect of these cytoplasmic mutations on shedding is likely due to the altered association of L-selectin with intracellular binding proteins.

Calmodulin (CaM), a small calcium-binding protein that ubiquitously interacts with many ligands in the cell, was the first reported intracellular regulator of L-selectin shedding [20]. Addition of membrane-permeable CaM inhibitors to cells expressing L-selectin induced shedding of L-selectin [20]. Since then, CaM inhibitors have been shown to induce shedding of many other membrane proteins [21]–[26]. CaM was shown to coimmunoprecipitate with full-length L-selectin in cell lysates and bind to the immobilized peptides corresponding to the cytoplasmic domain of L-selectin [15], [20]. It was hypothesized that CaM binds to the L-selectin cytoplasmic domain and inhibits shedding of L-selectin, and treatment of CaM inhibitors blocks CaM binding to the L-selectin cytoplasmic domain and therefore induces L-selectin shedding [20].

**Figure 1:**
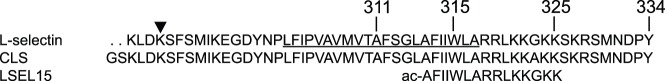
Sequences of L-selectin-derived peptides CLS and LSEL15. The sequence of C-terminal portion of human L-selectin, including entire transmembrane and cytoplasmic domains, is shown on top, with key residue numbers included. Residues in the transmembrane domain are underlined. The filled triangle marks the shedding cleavage site in L-selectin. The N-terminal end of LSEL15 is acetylated (ac-).

To test the so-called CaM hypothesis, we previously characterized the interaction of CaM with a recombinant fragment of human L-selectin named CLS under conditions mimicking the cell membrane environment [27], [28]. CLS contains both transmembrane and cytoplasmic domains of L-selectin (Fig. 1). When reconstituted in phospholipid liposomes, the transmembrane domain of CLS adopts α-helical structure that traverses the membrane bilayer, while its neighboring cytoplasmic domain is solvent-exposed and adopts a more flexible conformation [28]. Binding of CaM to CLS in liposomes is highly dependent on inclusion of negatively charged phosphatidylserine in the lipid bilayer [28]. In the absence of phosphatidylserine CaM binds to CLS in a Ca^2+^-independent manner with relatively weak affinity (*K*_d_ = 2 µM), which matches the characteristics of binding CaM to a water-soluble peptide containing L-selectin cytoplasmic residues Ala^317^–Tyr^334^. However, CaM does not bind to CLS when phosphatidylserine is included in the liposome to mimic the native lipid composition of the inner leaflet of the plasma membrane [28]. This is likely due to electrostatic interactions between negatively charged phosphatidylserine and basic residues in the cytoplasmic domain of CLS that sequester CLS from CaM [28].

Recently Gifford *et al.* [29] also examined interactions between CaM and several peptides derived from L-selectin in aqueous solutions. Consistent with our report, they found that CaM binds weakly to a peptide containing L-selectin residues Arg^318^–Tyr^334^, with a *K*_d_ around 10^−5^ M. However, complex binding behavior, with two binding events with *K*_d_ on the order of 10^−9^ M and 10^−6^ M, was observed for binding CaM to a peptide containing a portion of the transmembrane domain and the entire cytoplasmic domain of L-selectin (residues Ala^311^–Tyr^334^). Similar affinities were also obtained for the association of CaM with LSEL15, a peptide containing a portion of both transmembrane and cytoplasmic domains of L-selectin (residues Ala^311^–Lys^325^). Specifically, Ca^2+^-CaM binds to LSEL15 with a *K*_d_ of 43±37 nM.

The solution structure of the CaM/LSEL15 complex [29] showed that the two lobes of CaM wrapped around LSEL15 in a compact conformation that is similar to the structure of CaM bound to other high-affinity peptides [30]–[36]. Several residues in LSEL15, such as Ile^314^, Leu^316^ and Leu^320^, are involved in direct hydrophobic interactions with CaM. Since both residues Ile^314^ and Leu^316^ are located in the transmembrane domain of L-selectin, Gifford *et al*. suggested that a significant portion of the L-selectin transmembrane domain participates in direct association with CaM by hydrophobic interactions when CaM binds full-length L-selectin. This would necessitate a change in the association of the transmembrane domain with the lipid bilayer [29]. Although an interaction of CaM with a portion of L-selectin transmembrane domain would provide an attractive model to explain several features of L-selectin shedding, it appears contradictory to our earlier study of the association of CaM with CLS [28] because of the apparent mismatch of measured dissociation constants, and the lack of binding when CLS is associated with phosphatidylserine-containing liposome that mimics the cell membrane. To reconcile the difference in reported dissociation constants – 2 µM for CaM with CLS in the 1-palmitoyl-2-oleoyl-sn-glycero-3-phosphocholine (POPC) liposome [28] vs. 44 nM for CaM with LSEL15 in aqueous solution [29], Gifford *et al.* suggested a two-step model in which CaM first binds to the exposed L-selectin cytoplasmic domain with a micromolar dissociation constant via one domain to bring it into the proximity of the adjacent transmembrane domain. CaM then binds key residues in the transmembrane domain and transitions to a high-affinity compact complex in which both domains of CaM bind to L-selectin [29].

To test whether CaM adopts such a compact conformation when it binds to L-selectin in the membrane, we have characterized the conformation of CaM in complex with CLS in phospholipid liposomes. Our results reported in this paper demonstrate that CaM adopts an extended conformation that is consistent with the reported micromolar dissociation constant and distinctly different from that in the CaM/LSEL15 complex structure.

## Materials and Methods

### Materials

Preparation of CLS, CaM, IAEDANS-labeled CaM (I-CaM) and the CaM-binding peptide derived from CaMKII (FNARRKLKGAILTTMLATRN, residues 293–312) has been described before [27], [28]. Reconstitution of CLS into phospholipid liposomes and determination of its concentration in liposomes have also been described [27], [28]. Human moesin cDNA was a kind gift from Dr. Ronan Murphy. Synthetic lipids POPC and 1-palmitoyl-2-oleoyl-sn-glycero-3-phosphoserine (POPS) were purchased from Avanti Polar Lipids (Alabaster, AL). Peptide LSEL15 (ac-AFIIWLARRLKKAKK) was synthesized by Genscript (Piscataway, NJ) and further purified to 90–95% purity by reverse-phase HPLC. The extinction coefficient of LSEL15, 5,500 M^−1^ cm^−1^ at 280 nm, was estimated from the primary sequence using the method of Pace *et al.* [37].

### Expression and Purification of Moesin FERM Domain

The DNA fragment encoding human moesin FERM domain (residues 1–346) was amplified using primers 5′-GCATGAATTCCATGCCCAAAACGATC-3′ and 5′-GCATCTCGAGTCACTCCTCCTTCTCCCGTTC-3′, and subcloned as an EcoR1/Xhol fragment into the pHex vector [38]. Expression of the glutathione s-transferase (GST)-moesin fusion protein in *E. coli* BL21 cells was induced by 1 mM IPTG at 37°C for 3 hours. To purify the fusion protein, the cell pellet was suspended in 50 mM Tris, 500 mM NaCl, 1 mM dithiothreitol (DTT), pH 7.4 buffer that contained 1 mM phenylmethylsulfonyl fluoride and lysed by sonication on ice. After centrifugation, the supernatant was loaded onto a glutathione sepharose 4B column (GE Healthcare Biosciences, Pittsburgh, PA). The GST-moesin fusion protein was eluted with 50 mM Tris, 500 mM NaCl, 20 mM reduced glutathione, pH 8.0 before being mixed with thrombin at 4 u/mg of fusion protein and dialyzed overnight against 50 mM Tris, 150 mM NaCl, 1 mM DTT, pH 7.4. The mixture was then loaded onto the re-equilibrated glutathione sepharose 4B column to remove all the GST-containing fragments. The moesin FERM domain was further purified by gel filtration chromatography. Its purity was confirmed by SDS-PAGE. Its concentration was measured using the extinction coefficients of 50,800 M^−1^ cm^−1^ at 280 nm [37]. The protein stock was stored at −80°C before use.

### Fluorescence Titration of LSEL15 and CaM

To follow the steady-state fluorescence change of Trp^315^ in LSEL15 in response to titration of unlabeled CaM, LSEL15 was dissolved in 2.0 ml of 10 mM MOPS buffer, pH 7.4, containing 100 mM NaCl, 0.3 mM CaCl_2_ and 0.1 mg/ml bovine serum albumin (BSA), to achieve a final concentration of approximately 1 nM. The CaM solution was prepared with the same buffer containing LSEL15 so the LSEL15 concentration was kept constant during the titration. All solutions were filtered prior to the experiments. CaM was titrated into LSEL15 solution and the emission was acquired on a PTI QuantaMaster spectrometer (Photon Technology International, Birmingham, NJ) using a 3-ml cuvette. The excitation wavelength was set to 295 nm. The titration was repeated 3 times independently to obtain the averaged emission intensity. The slit widths for excitation and emission were adjusted to minimize photo bleaching of the sample and to achieve sufficient fluorescent signal intensity. When applicable, the fluorescence measurements as a function of peptide concentration were fitted with the hyperbolic binding equation as described [28].

IAEDANS fluorescence was followed to measure the titration of LSEL15 to I-CaM. Briefly, the stock solution of I-CaM was dissolved to 2 ml of the same buffer as above to achieve a final protein concentration of approximately 1 nM. LSEL15 solution was prepared in the same buffer containing I-CaM so that the I-CaM concentration was kept constant during titration. The IAEDANS emission fluorescence was acquired on the same instrument with the excitation wavelength at 340 nm.

### Fluorescence Resonance Energy Transfer (FRET) Measurements

CLS was reconstituted into the phospholipid liposome as described before [28]. The molar ratio of CLS to POPC was kept as 1∶1000 in this study. Both LSEL15 and CLS reconstituted in the POPC liposome (CLS/POPC) were dissolved in 10 mM MOPS, pH 7.4 buffer that contained 100 mM NaCl and 0.3 mM CaCl_2_ to achieve a final concentration of approximately 26 nM for the measurement. Each sample was incubated at 25°C for at least 5 minutes; and the fluorescence spectra were collected multiple times to confirm that the equilibrium had been reached. The Trp^315^ emission fluorescence spectra at 310–400 nm were recorded with the excitation wavelength set to 295 nm. The spectra were collected and corrected with background fluorescence from the buffer. The FRET efficiency (*E*) was calculated by using equation 1,
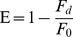
(1)where *F*_0_ and *F*_d_ are the Trp emission fluorescence intensities without and with I-CaM, respectively. The distance between donor and acceptor (*r*) was calculated from equation 2,
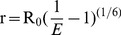
(2)where R0, the Förster radius for the Trp/IAEDANS pair, is assumed to be 22 Å [39]–[41].

### Fluorescence Quenching of D/A-CaM

Emission fluorescence of D/A-CaM was measured essentially as described before [28]. The sample buffer was the same as in the FRET experiment. All spectra were corrected with background fluorescence from the buffer.

### Time-based IAEDANS Fluorescence Emission Measurement

I-CaM stock was diluted into 2 ml of 10 mM MOPS, pH 7.4 buffer that contained 100 mM NaCl, 0.3 mM CaCl_2_ to achieve a final concentration of approximately 125 nM. Both moesin FERM domain and CLS/POPC in the same buffer were added sequentially into the I-CaM solution to a final concentration of 10 µM at indicated time points. The IAEDANS emission fluorescence was recorded over time with excitation and emission wavelengths set to 340 and 475 nm, respectively. The scanning speed was once per second.

## Results

### Differential FRET between L-selectin Fragments and I-CaM in Membrane and Aqueous Conditions

To directly compare the conformation of CaM in its complex with LSEL15 in the aqueous solution and that in its complex with CLS (*i.e.* entire transmembrane and cytoplasmic domains of L-selectin) in the membrane bilayer, we chose two methods, both of which utilize fluorescent probes and can be applied in both aqueous and membrane conditions. Although the CaM/LSEL15 complex is present in aqueous environment and the CaM/CLS complex in a membrane environment, it should be emphasized that the fluorescent probes used were the same in both complexes.

The first method is to measure the fluorescence resonance energy transfer (FRET) between L-selectin residue Trp^315^ and the IAEDANS group attached to residue 75 of CaM. Trp^315^ is the only Trp residue in LSEL15 and CLS, and CaM has no Trp residues. Trp^315^ is located near the C-terminal end of the L-selectin transmembrane domain (Fig. 1). Lys^75^ is located in the linker region between the two lobes of CaM. Upon ligand binding the linker region generally undergoes a significant conformational change as the two lobes of CaM wrap around the ligand, but it does not make direct contact with the ligand [42]. The IAEDANS-labeled K75C mutant CaM, termed I-CaM, has been used to follow the interaction of CaM with its peptide ligands [42]–[44]. The IAEDANS group does not interfere with the interaction of CaM with CLS [28].

**Figure 2:**
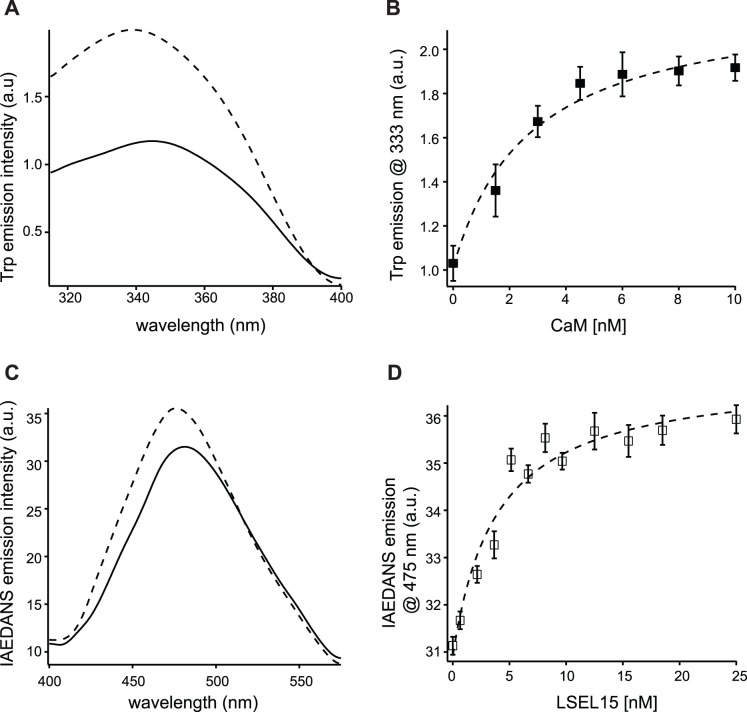
The association of I-CaM with LSEL15 in aqueous solution. A, Trp fluorescence emission spectra of 1 nM LSEL15 in the absence (solid line) and presence (dash line) of 10 nM Ca^2+^-loaded CaM. LSEL15 and CaM were dissolved in 2 ml of 10 mM MOPS, 0.3 mM CaCl_2_, 100 mM NaCl, 0.1 mg/ml BSA, pH 7.4. The emission spectra were obtained with the excitation wavelength at 295 nm and each was the average of 3 scans. Both spectra were corrected for background fluorescence from the buffer. B, the titration plot of LSEL15 by Ca^2+^-CaM. CaM was titrated to the LSEL15 solution at indicated concentrations, and the Trp emission at 333 nm was measured. Dashed line indicates the fitted binding curve. C, IAEDANS fluorescence emission spectra of 1 nM Ca^2+^-I-CaM in the absence (solid line) and presence (dash line) of 25 nM LSEL15. I-CaM and LSEL15 were dissolved in 2 ml of the same buffer as in (A). The emission spectra were obtained with the excitation wavelength at 340 nm and each was the average of 3 scans. Both spectra were corrected for background fluorescence from the buffer. D, the titration plot of Ca^2+^ loaded I-CaM by LSEL15. LSEL15 was titrated to the I-CaM solution at indicated concentration, and the IAEDANS emission at 475 nm was measured. Dashed line indicates the fitted binding curve.

To test whether attachment of an IAEDANS probe to CaM significantly alters its association with LSEL15, we measured the dissociation constants of LSEL15 to both unlabeled CaM and I-CaM in 10 mM MOPS, pH 7.4 buffer containing 0.3 mM CaCl_2_ and 100 mM NaCl. As shown in Figure 2A, the emission fluorescence of Trp^315^ increased with titration of unlabeled CaM to LSEL15. The maximal emission wavelength was concurrently blue-shifted, which typically indicated the occurrence of hydrophobic interactions [45], [46]. The plot of Trp emission fluorescence as a function of CaM concentration fitted well to the hyperbolic binding equation with a dissociation constant of 2.7±0.9 nM (Fig. 2B). When LSEL15 was mixed with I-CaM, the Trp emission fluorescence was quenched by the IAEDANS group. Thus, the association of LSEL15 to I-CaM was monitored instead through the steady-state IAEDANS fluorescence. Upon the titration of LSEL15, an increase in IAEDANS emission fluorescence, coupled with a blue-shift in maximal emission wavelength, was observed (Fig. 2C). Fitting the titration plot produced a dissociation constant of 3.8±0.2 nM (Fig. 2D). The dissociation constants obtained for the CaM/LSEL15 and I-CaM/LSEL15 associations were essentially the same, and they were in agreement with that reported by Gifford *et al.* [29] for the CaM/LSEL15 association. Thus, we concluded that the attached IAEDANS probe did not affect the association of CaM with LSEL15.

**Figure 3:**
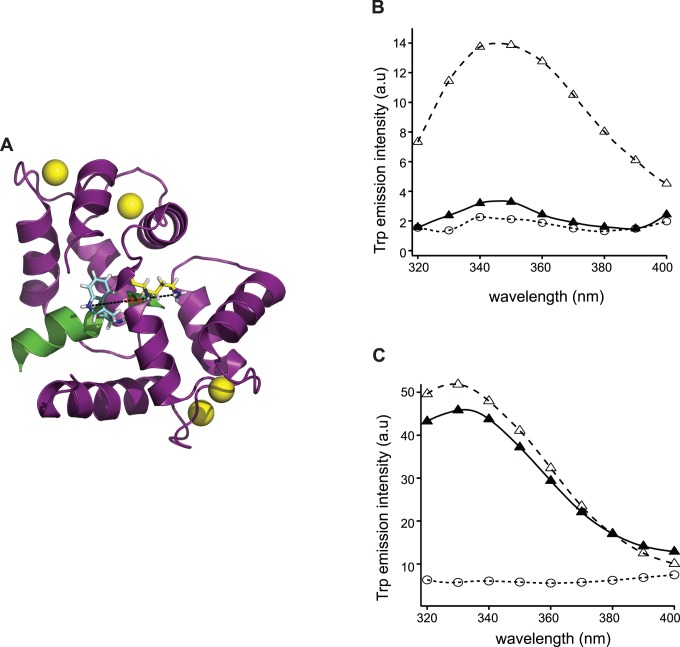
Differential FRET between L-selectin fragments and CaM in membrane and aqueous conditions. A, a ribbon diagram showing the proximity of Trp^315^ of LSEL15 and Lys^75^ of CaM in the CaM/LSEL15 complex structure (PDB ID: 2LGF). The LSEL15 peptide is shown in green, and CaM in purple. Four calcium ions are shown as yellow spheres. The side chains of Trp^315^ and Lys^75^ are shown in sticks, with the dashed line connecting the Nε1 atom in Trp^315^ and the Nζ atom in Lys^75^. B, Trp emission spectra of 26 nM LSEL15 mixed with 104 nM unlabeled CaM in 10 mM MOPS, 100 mM NaCl, 0.3 mM CaCl_2_, pH 7.4 (▵), of 104 nM I-CaM alone in the same buffer (○) and of 26 nM LSEL15 mixed with 104 nM I-CaM in the same buffer (▴). C, Trp emission spectra of 26 nM CLS/POPC (1/1000 molar ratio) mixed with 104 nM unlabeled CaM in the same buffer as in (B) (▵), of 10 µM I-CaM alone with empty POPC liposome (○), and of 26 nM CLS/POPC mixed with 10 µM I-CaM (▴). All emission spectra were obtained with the excitation wavelength at 295 nm and each was the average of 3 scans. All spectra were corrected for background fluorescence from the buffer.

To determine the FRET efficiency between Trp^315^ and IAEDANS in the I-CaM/LSEL15 complex, 26 nM LSEL15 was mixed with 104 nM unlabeled CaM to produce the donor-only Trp emission spectrum. 104 nM I-CaM alone was used to produce the acceptor-only spectrum. The mixture of 26 nM LSEL15 and 104 nM I-CaM in the same buffer was used to produce the donor/acceptor spectrum (Fig. 3B). Each sample was prepared and incubated at room temperature for at least 5 minutes before the steady-state spectrum was collected. As shown in Figure 3B, the emission of Trp^315^ in LSEL15 was significantly quenched by the bound I-CaM, indicating the close proximity of Trp^315^ to the IAEDANS group in the I-CaM/LSEL15 complex. The FRET efficiency was measured to be 0.93±0.04. With the assumptions that both probes can undergo unrestricted motion and the Förster radius for the Trp/IAEDANS pair is 22 Å [40], [41], [47], the distance between the two probes was calculated as 14.3±2.0 Å. This is in excellent agreement with the NMR structure of the CaM/LSEL15 complex [29], as the distance between the Nε1 atom of Trp^315^ in LSEL15 to the Nζ atom of Lys^75^ in CaM is 12.4 Å (Fig. 3A).

Since CaM does not associate with CLS when CLS is embedded in the membrane containing negatively charged phosphatidylserine lipid [28], the analysis of the CaM/CLS complex was carried out here in POPC liposomes. We had previously shown that I-CaM bound to CLS/POPC with a dissociation constant of approximate 2.0 µM and in a calcium-independent manner [28]. To determine the FRET efficiency in the I-CaM/CLS complex, 26 nM CLS/POPC (at 1∶1000 protein/lipid molar ratio) was mixed with 10 µM CaM and I-CaM to produce the donor-only and donor/acceptor spectra, respectively. I-CaM mixed with 26 µM empty POPC liposome was used to produce the acceptor-only spectrum. Comparison of these Trp emission spectra showed that the emission of Trp^315^ in CLS was quenched slightly by I-CaM (Fig. 3C). The observed FRET efficiency was 0.22±0.02. Due to the limited availability of I-CaM, 10 µM instead of a higher concentration was used in this measurement. With a dissociation constant of approximate 2.0 µM [28], it was expected that 83% of CLS/POPC were bound to I-CaM in this experiment. Assuming there is no FRET between I-CaM and unbound CLS, the actual FRET efficiency of Trp^315^ and IAEDANS in the I-CaM/CLS/POPC complex should be adjusted to 0.26±0.03, which corresponds to a distance of 26.0±0.5 Å between the two probes. This distance is significantly longer than that between the same groups in the I-CaM/LSEL15 complex, indicating that CaM in the CaM/CLS/POPC complex adopts a distinctly different conformation from that in the CaM/LSEL15 complex.

### Fluorescence Quenching of D/A-CaM is Different between CaM/CLS/POPC and CaM/LSEL15 Complexes

The second method is to directly assess whether CaM adopts an extended or a more compact conformation when it binds to various L-selectin fragments. Upon ligand binding, the two lobes of CaM often wrap around the ligand and become closer in space [31], [48]–[50]. In the T34C/T110C mutant CaM, a fluorescence donor (IAEDANS) and a non-fluorescent acceptor N-(4-dimethylamino-3,5-dinitrophenyl) maleimide (DDPM) can be simultaneously attached to the two lobes [51]. Thus, the extent of fluorescence quenching in the donor/acceptor double-labeled CaM (D/A-CaM) can be used to monitor the spatial distance between the two lobes of CaM.

In the CaM/LSEL15 complex structure, the two lobes of CaM wrap tightly around LSEL15 [29]. The distance between the Cγ atom of Thr^34^ in the N-lobe and the Cγ atom of Thr^110^ in the C-lobe is 13.6 Å, which is similar to the 13.8-Å distance between the same atoms in the crystal structure of CaM in its complex with a peptide corresponding to residues 293–312 of CaM-dependent protein kinase II (CaMKII) [31].

**Figure 4:**
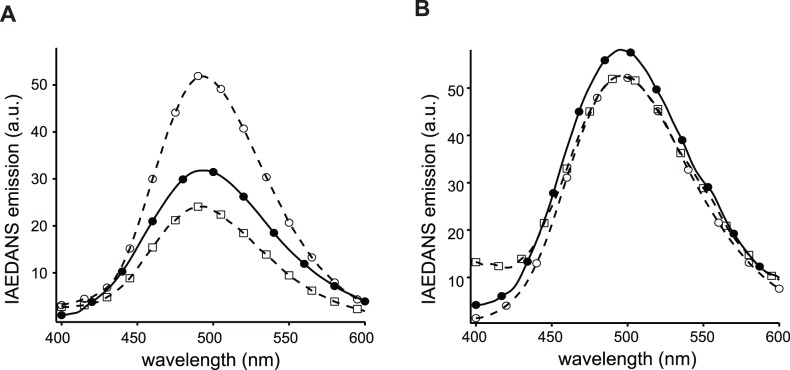
Fluorescence quenching in D/A-CaM is different in CaM/CLS/POPC and CaM/LSEL15 complexes. A, IAEDANS emission spectra of 30 nM D/A-CaM in 10 mM MOPS, 100 mM NaCl, 0.3 mM CaCl_2_, pH 7.4 (○), and of 30 nM D/A-CaM in the presence of 100 nM LSEL15 (•) or 100 nM CaMKII peptide (□). B, IAEDANS emission spectra of 30 nM D/A-CaM in buffer (○), in the presence of empty POPC liposome (□) and in the presence of 10µM CLS/POPC (•). All emission spectra were obtained with the excitation wavelength at 340 nm and each was the average of 3 scans. All spectra were corrected for background fluorescence from the buffer.

To assess the spatial closeness of the two lobes of CaM in complex with LSEL15, 30 nM D/A-CaM was mixed with either 100 nM LSEL15 or only the buffer. Each sample was incubated at room temperature for at least 5 minutes to ensure that equilibrium had been reached before data collection. Since the dissociation constant of the CaM/LSEL15 complex was around 3 nM (Fig. 2), all of D/A-CaM in the mixture was expected to be in the LSEL15-bound form. The IAEDANS emission fluorescence was recorded, corrected for background fluorescence and compared with that from 30 nM D/A-CaM mixed with 100 nM CaMKII-derived peptide. As shown in Figure 4A, addition of LSEL15 to D/A-CaM induced significant quenching of the IAEDANS fluorescence, the extent of which was similar to that induced by the CaMKII-derived peptide. This result indicated that when it associates with LSEL15 peptide CaM takes on a compact conformation, which is similar to that with the CaMKII-derived peptide.

The spatial distance between the two lobes of CaM in its complex with CLS/POPC was evaluated in a similar way. The IAEDANS emission fluorescence of 30 nM D/A-CaM was measured in the presence of empty POPC liposome or 10 µM CLS/POPC. As shown in Figure 4B, addition of CLS/POPC to D/A-CaM did not induce any quenching of the IAEDANS fluorescence. Instead, the fluorescence intensity of IAEDANS increased slightly, suggesting that the two lobes of CaM stay farther apart in its complex with CLS/POPC, in contrast to the CaM/LSEL15 complex.

In summary, the measurements of both FRET and D/A-CaM quenching showed that CaM adopts a compact conformation with its two lobes wrapping around LSEL15 in aqueous solutions, which is consistent with the NMR structure of the CaM/LSEL15 complex. In contrast, when CaM associates with CLS in POPC liposomes, it adopts a distinctly different and more extended conformation, with its two lobes apart from each other. CLS contains the entire transmembrane and cytoplasmic domains of L-selectin and therefore resembles more closely full-length L-selectin in the membrane than LSEL15 does in aqueous solution. Our results have demonstrated clearly that the conformation of CaM in complex with LSEL15 as in the NMR structure reported by Gifford *et al.* [29] does not represent that in complex with L-selectin in a membrane bilayer.

### Fluorescence Quenching of D/A-CaM in a Ternary Complex with Moesin FERM Domain and CLS in Membranes

CaM can form a ternary complex with moesin and L-selectin, and this ternary complex was proposed to participate in the regulation of L-selectin shedding [52]. Killock *et al.* further proposed a structural model for the ternary complex, in which CaM adopted an extended conformation and mostly interacted with the L-selectin cytoplasmic domain via its C-lobe [52]. Based on the CaM/LSEL15 complex structure, Gifford *et al.* also generated a structural model for this ternary complex in which CaM adopts a compact conformation and interacts with transmembrane residues of L-selectin [29]. To test whether CaM adopts an extended or a compact conformation in the ternary complex, we measured the fluorescence quenching of D/A-CaM when it formed a ternary complex with the moesin FERM domain and CLS in liposomes that mimic the native membrane environment.

**Figure 5:**
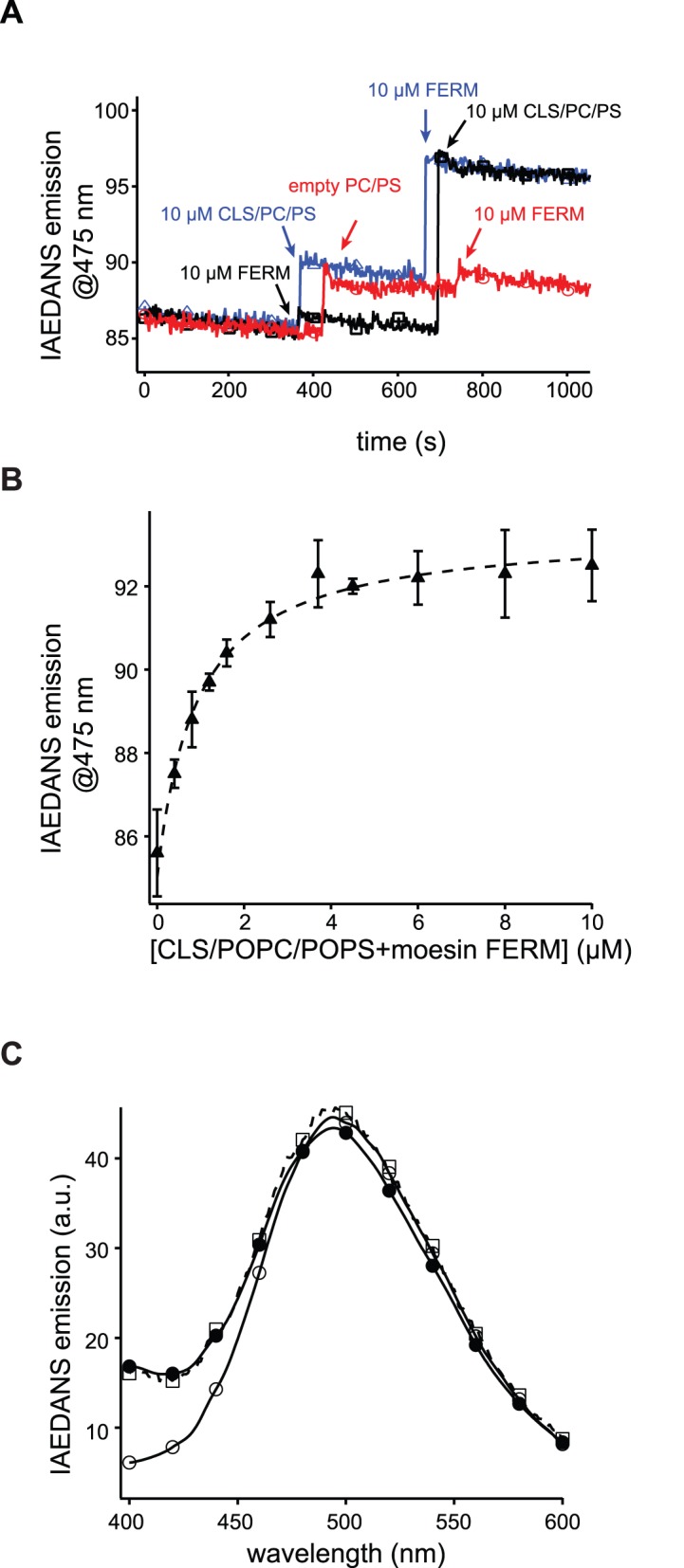
CaM adopts an extended conformation in its ternary complex with CLS and moesin FERM domain. A, time-based IAEDANS emission traces of 125 nM I-CaM in 10 mM MOPS, 100 mM NaCl, 0.3 mM CaCl_2_, pH 7.4. 10 µM (final concentration) moesin FERM domain or 10 µM (final concentration) CLS/POPC/POPS (1/850/150 molar ratio) or empty POPC/POPS liposome was added to the I-CaM solution at indicated time points. Differently colored traces indicate separate runs. The excitation and emission wavelengths were set to 340 and 475 nm, respectively. B, The titration plot of I-CaM by the complex of CLS/POPC/POPS and moesin FERM domain. The mixture of CLS/POPC/POPS and moesin FERM, at 1∶1 molar ratio, was titrated to the I-CaM solution at indicated concentrations, and the IAEDANS emission at 475 nm was measured. The background scattering fluorescence as a result of added liposome was subtracted from each measurement. Dashed line indicates the fitted binding curve. C, IAEDANS fluorescence emission spectra of 30 nM D/A-CaM in the buffer (○), of 30 nM D/A-CaM mixed with empty POPC/POPS liposome (□), and of 30 nM D/A-CaM mixed with 10 µM CLS/POPC/POPS (1/850/150 molar ratio) and 10 µM moesin FERM domain (•). All emission spectra were obtained with the excitation wavelength at 340 nm and each was the average of 3 scans. All spectra were corrected for background fluorescence from the buffer.

Figure 5A shows formation of the ternary complex in 10 mM MOPS, pH 7.4 buffer that contained 100 mM NaCl and 0.3 mM CaCl_2_ through time-based IAEDANS emission fluorescence of I-CaM at 475 nm. When 30 nM I-CaM was mixed with 10 µM CLS/POPC/POPS (1/850/150 molar ratio), only the nonspecific fluorescence increase, due to scattering of the added liposome, was observed, which is consistent with our earlier finding that CaM does not associate with CLS in PS-containing liposomes [28]. Similarly, addition of 10 µM moesin FERM domain to the I-CaM solution did not induce a specific change in the IAEDANS fluorescence, indicating that moesin FERM domain does not associate with CaM. However, adding both moesin FERM domain and CLS/POPC/POPS induced a significant increase in the IAEDANS fluorescence, indicating the formation of the CaM/moesin-FERM/CLS ternary complex on the surface of the POPC/POPS liposome (Fig. 5A). Further characterization of the association indicated that the apparent dissociation constant of I-CaM with the CLS/moesin-FERM complex was approximately 1 µM (Fig. 5B). Thus, in the presence of 10 µM moesin FERM domain and CLS/POPC/POPS more than 90% of I-CaM was in the ternary complex.

To characterize the conformation of CaM in the ternary complex, D/A-CaM instead of I-CaM was mixed with 10 µM moesin FERM domain and CLS/POPC/POPS in the aforementioned MOPS buffer, and the IAEDANS emission fluorescence was recorded and compared to that of D/A-CaM alone in the MOPS buffer and in the presence of empty POPC/POPS liposomes. As shown in Figure 5C, the IAEDANS fluorescence was quenched only slightly upon D/A-CaM association with CLS and moesin FERM domain. This result indicated that, in its ternary complex with moesin FERM domain and CLS, CaM adopts an extended conformation with its two lobes apart from each other. Such extended conformation is consistent with the model proposed by Killock *et al*. [52], but not with that proposed by Gifford *et al.* [29].

## Discussion

This study used two independent FRET methods to characterize and compare the conformation of CaM when bound to peptides derived from L-selectin. The characteristics of CaM association with LSEL15 are similar to those seen for peptides derived from proteins such as CaMKII and myosin light chain kinase. Specifically, CaM binds to LSEL15 in the presence of Ca^2+^, and the conformation of bound CaM is compact with the N- and C-lobes in close proximity to each other (Figs. 3 and 4). These properties are consistent with the solution structure of the Ca^2+^-CaM/LSEL15 complex [29] in which the N- and C-lobes of CaM bind to adjacent sites on LSEL15 via hydrophobic residues that include Ile^314^ and Leu^316^ in the transmembrane domain of L-selectin. In contrast, the same FRET measurements on the CaM complex with CLS in membrane bilayers showed that CaM remains in an extended conformation (Figs. 4 and 5). Furthermore, CaM also adopts an extended conformation in the Ca^2+^-CaM/CLS/moesin ternary complex, which is distinctly different from that in the Ca^2+^-CaM/LSEL15 complex (Fig. 5). Since L-selectin is an integral membrane protein and the CaM-binding site is located close to its transmembrane domain, CLS reconstituted in the phospholipid membrane resembles full-length L-selectin in the cell membrane more than LSEL15 does in the aqueous solution. Thus, the structure of the Ca^2+^-CaM/LSEL complex is unlikely to represent that of the CaM/L-selectin association in the membrane. The contrasting results obtained for the CaM/CLS complex in the phospholipid membrane versus those for the CaM/LSEL15 complex in the aqueous solution highlight the importance of studying such interactions under appropriate native-like settings.

A large number of studies have led to the current understanding of CaM association with its ligands. Calcium binding to CaM induces formation of hydrophobic clefts in both N- and C-lobes that can in turn bind closely with hydrophobic residues in the ligand, thus greatly enhancing its binding affinity [49], [53]. Connected by a central flexible linker, both lobes of CaM can wrap around its peptide ligands in different ways, yet still achieving high binding affinity [54]–[56]. Thus, in the case of CaM association with LSEL15, the presence of calcium ion in the complex structure, the nanomolar dissociation constant, and the close proximity between the two lobes of CaM are all measurable signs of the direct interaction between CaM and hydrophobic residues from the L-selectin transmembrane domain such as Ile^314^ and Leu^316^. By comparison, the association of CaM with CLS in the membrane is characterized by a lack of dependence on calcium ion, the relatively low micromolar dissociation constant [28], and a wide separation between the two lobes of CaM as shown in this study (Fig. 4, 5). Therefore, we conclude that CaM does not bind directly to L-selectin transmembrane residues such as Ile^314^ and Leu^316^ when it associates with CLS in the phospholipid bilayer.

**Figure 6:**
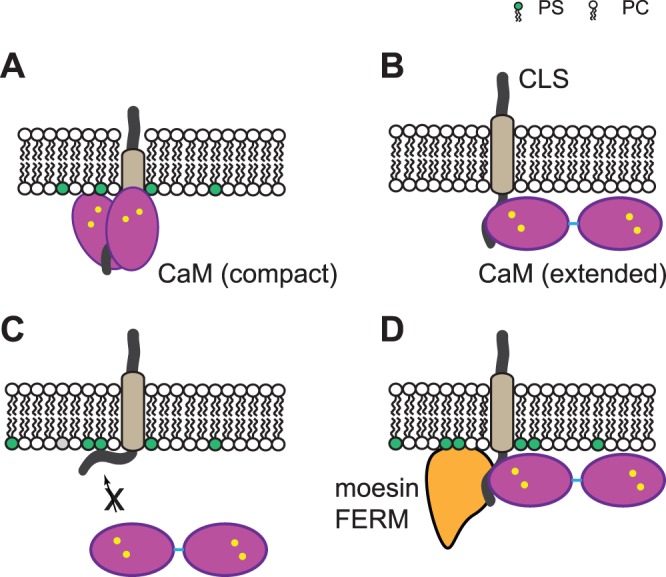
Illustrations of CaM association with L-selectin fragments. Gifford *et al.* [29] proposed that (A) calcium-loaded CaM associates with part of the L-selectin transmembrane domain and takes on a compact conformation. However, we have found that (B) CaM adopts an extended conformation when it binds the cytoplasmic domain of CLS in the membrane bilayer. (C) When phosphotidylserine (PS) is in the bilayer, the L-selectin cytoplasmic domain interacts with the negatively charged PS and not with CaM. (D) In the ternary complex of moesin FERM domain, CaM and CLS in the membrane, CaM still adopts an extended conformation.

The simplest explanation for the difference between CaM/LSEL15 and CaM/CLS complexes is that exposure of hydrophobic residues such as Ile^314^ and Leu^316^ in aqueous LSEL15 makes them available for association with Ca^2+^-CaM. Since these residues are part of the transmembrane helix, they are likely buried in the membrane bilayer and shielded from CaM when CLS is inserted into the liposome. Importantly, since CaM has an extended conformation when bound to CLS, these residues likely remain out of contact with CaM even when CaM becomes associated with the adjacent cytoplasmic domain of liposome-associated CLS (Fig. 6). This is inconsistent with the model of Gifford *et al.* that CaM first binds to cytoplasmic residues of L-selectin and can then “sense” key residues in the neighboring transmembrane helix and “pull” them out of the membrane [29] (Fig. 6A). The malleable membrane bilayer can, to a certain extent, be compressed or stretched along the membrane normal by hydrophobic matching to accommodate a shorter or longer transmembrane helix [57], [58], but this process costs free energy. In the case of CaM “pulling” L-selectin, it would cost even more free energy since the transfer of Ile^314^ and Leu^316^ out of the hydrophobic environment of the membrane bilayer is coupled to the thermodynamically unfavorable insertion of polar or charged residues into the membrane bilayer from the extracellular side as well as the extensive contact of the hydrophilic surface of CaM with the hydrophobic membrane bilayer on the cytoplasmic side. Finally, in general it takes more free energy to partition a portion of the transmembrane helix from a thicker membrane bilayer into the aqueous phase than from a thinner membrane bilayer. The hydrophobic bilayer of the POPC membrane is around 27 Å thick, which is thinner than that of an average mammalian plasma membrane by about 10–15 Å [59]–[62]. Therefore, it would take even more free energy (*i.e.* less likely) for CaM to pull a portion of the transmembrane domain of L-selectin out of the plasma membrane in a cell.

The CaM hypothesis stipulates that CaM binds to L-selectin and inhibits its shedding when the cell is not activated [20]. When the cell is not activated, the cytoplasmic Ca^2+^ concentration is low and the inner leaflet of the plasma membrane is enriched with phosphatidylserine lipids. We had shown previously that while CaM binds to CLS/POPC in a Ca^2+^-independent manner, it does not bind directly to CLS/POPC/POPS when the POPS content mimics that in the native plasma membrane [28] (Fig. 6C). These results suggest that CaM is not capable of association with L-selectin alone in the unstimulated cell. We further demonstrated here that, even when associating with CLS in the liposome, CaM adopts an extended conformation and does not interact with the transmembrane domain of L-selectin. Thus, it is unlikely that binding of CaM alone induces a movement of the L-selectin transmembrane domain across the cell membrane as suggested by Gifford *et al.* [29]. How CaM regulates shedding of L-selectin remains to be elucidated. One possibility is that CaM associates with L-selectin through another intracellular protein such as moesin [52]. This is consistent with earlier reports that mutations in the juxtamembrane region of the L-selectin cytoplasmic domain disrupt the phorbol ester-induced shedding of L-selectin [18], [19]. Although we showed in this study that CaM adopts an extended conformation in its complex with the moesin FERM domain and CLS in liposomes (Fig. 6D), it remains to be determined whether CaM modulates the interaction between the moesin FERM domain and the L-selectin cytoplasmic domain and whether such modulation helps to keep L-selectin in a shedding-resistant state. Nonetheless, considering the proximity of such ternary complex to the membrane surface, it will be imperative that future investigation be conducted in a membrane environment.
